# Supplementary material for: Discovery of immunotherapy targets for pediatric solid and brain tumors by exon-level expression
Source: Nat Commun. 2024 May 3;15:3732. doi: 10.1038/s41467-024-47649-y (PMC11068777; doi:10.1038/s41467-024-47649-y)
Supplement: Supplementary file 1 — Supplementary Information [file 41467_2024_47649_MOESM1_ESM.pdf]

Supplementary Figure 1

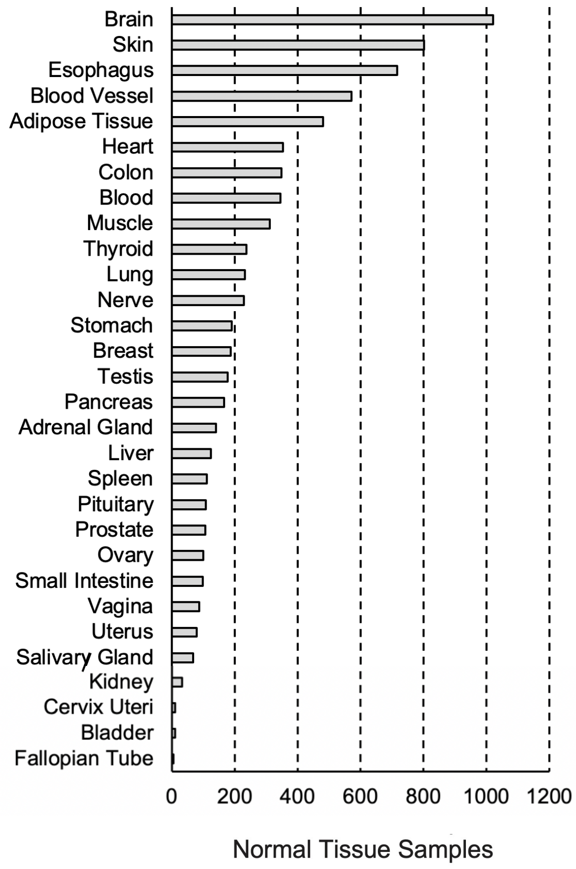

Supplementary Figure 1. Normal tissue types and their distribution in 7,460 RNA-seq data downloaded from GTEx portal version 7. Supplementary reference: 1.

Supplementary Figure 2

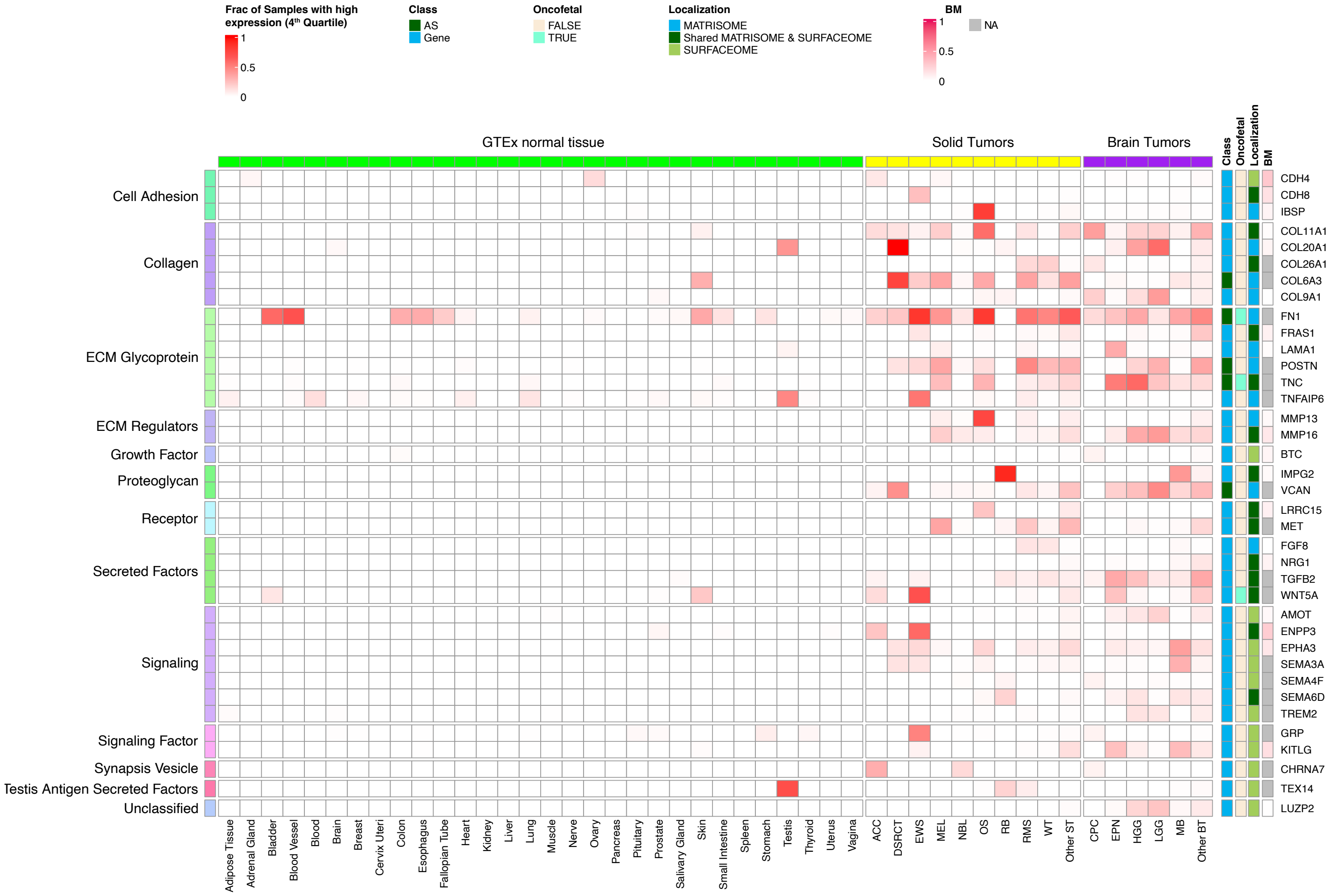

Supplementary Figure 2. Prevalence of samples with high expression of Tier 1 targets. Heatmap has the same layout as Figure 2; with the color scale represents the proportion of samples shown high expression (4th quartile) in a tumor type or in a normal tissue type. Expr: expression. BM: bone marrow. NA: not available. GTEx normal tissue: n=7,460; tumor samples: n=1,532. Supplementary references: 1, 2, 3, 4.

Supplementary Figure 3

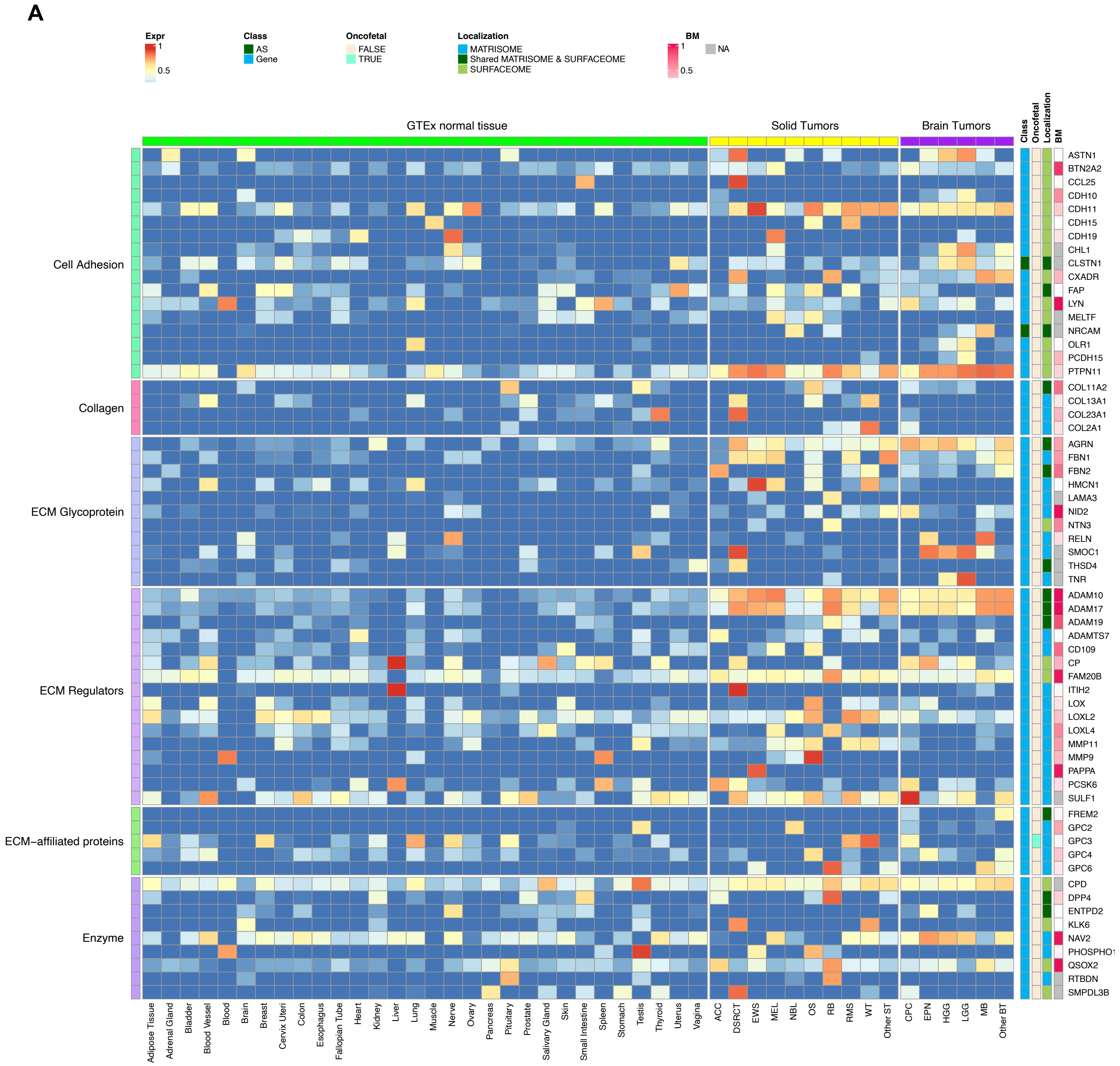

Supplementary Figure 3 (continued)

B

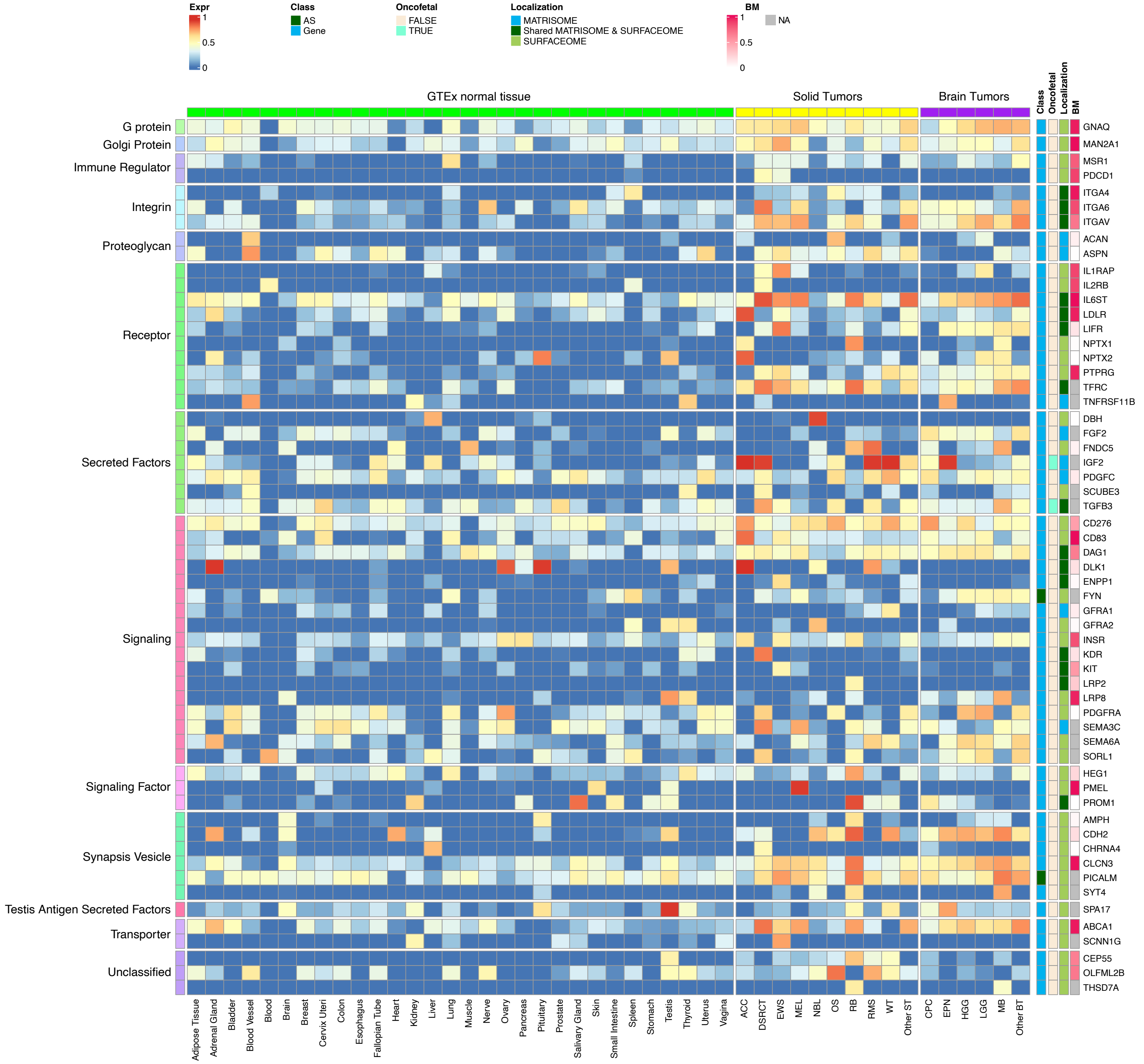

Supplementary Figure 3. Normalized expression of Tier 2 targets across pediatric cancer types and normal tissues. The heatmap spilt into panels A and B has the same layout as Figure 2. Expr: expression. BM: bone marrow. NA: not available. GTEx normal tissue: n=7,460; tumor samples: n=1,532. Supplementary references: 1, 2, 3, 4.

Supplementary Figure 4

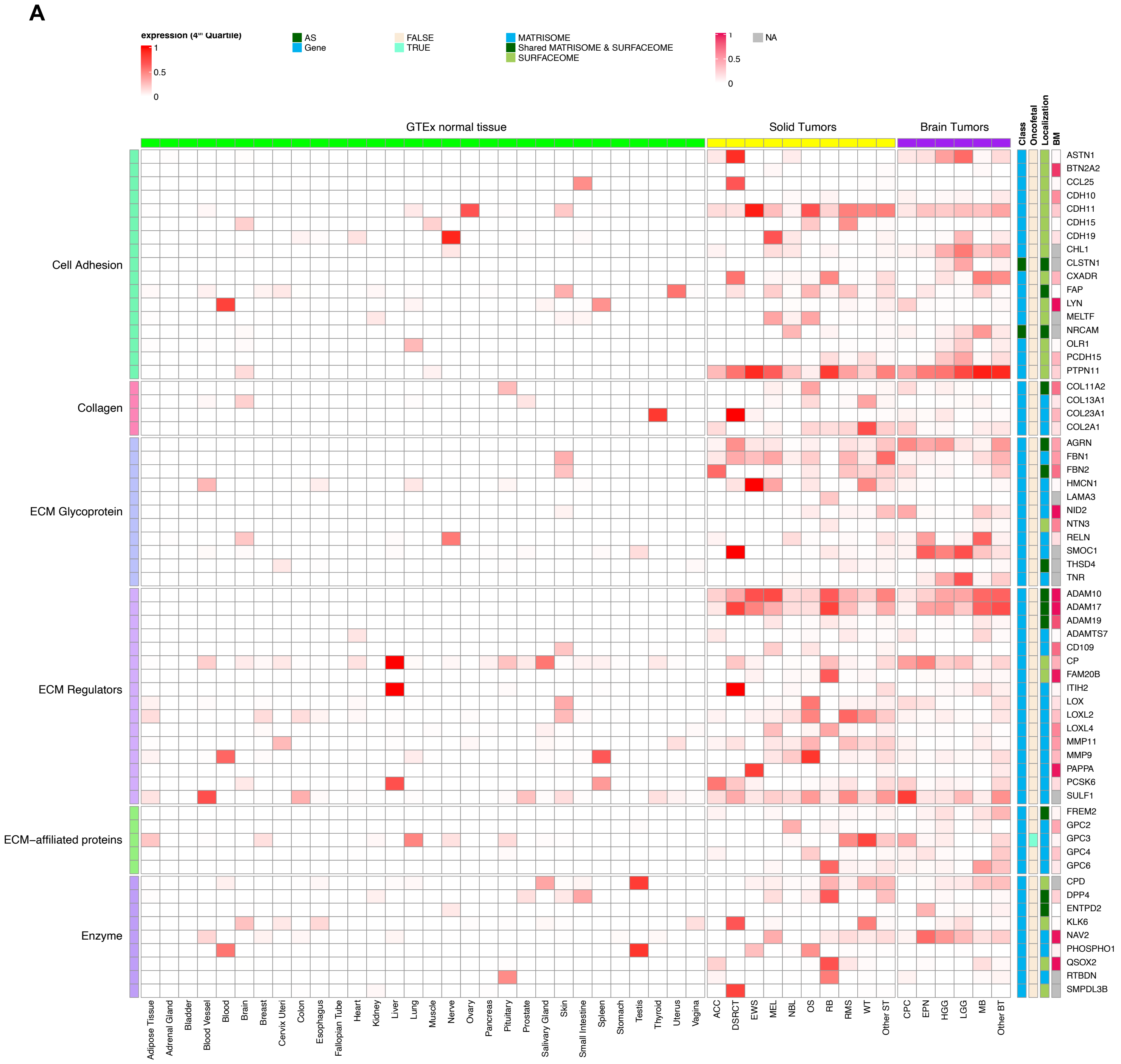

Supplementary Figure 4 (continued)

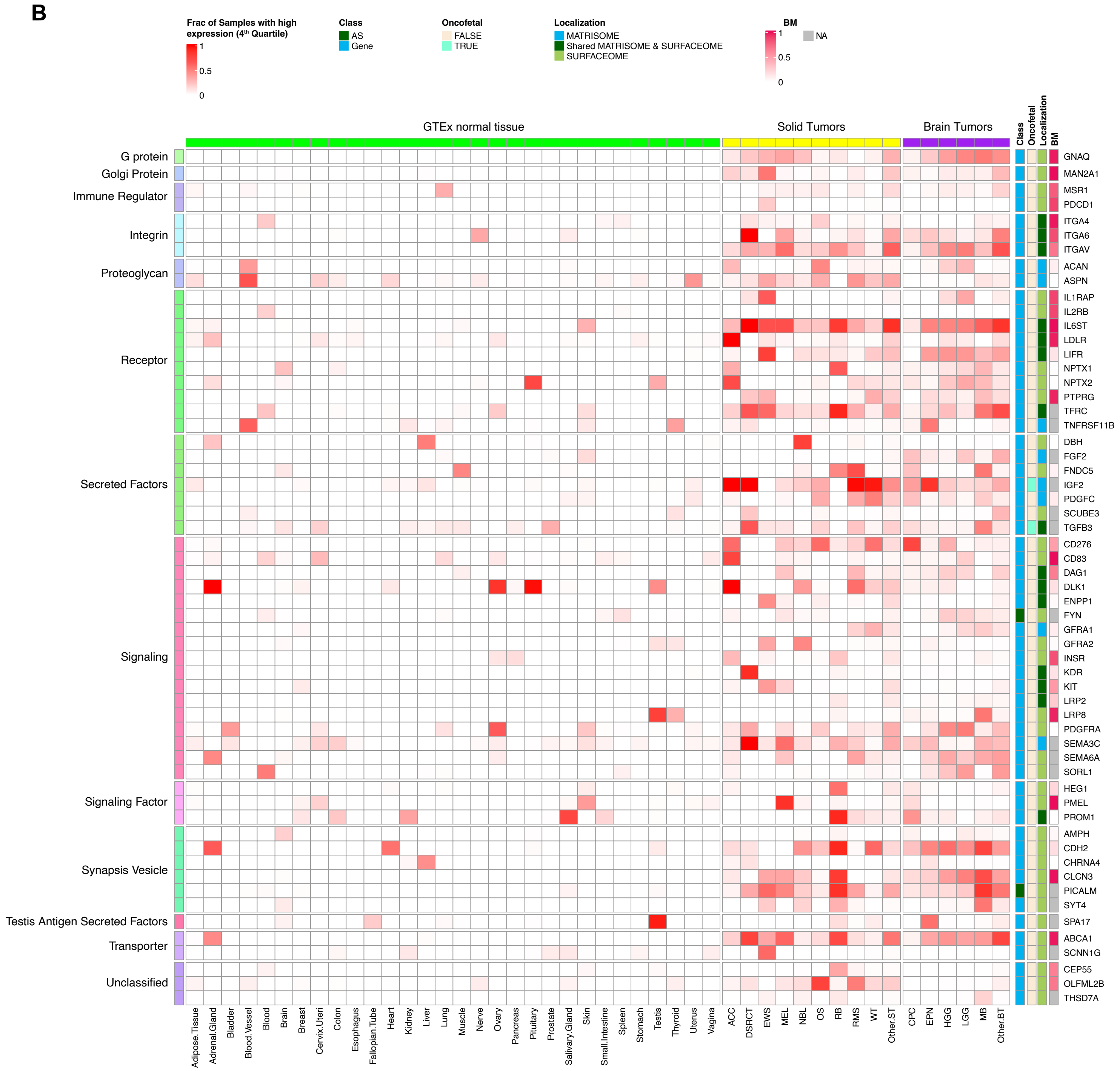

Supplementary Figure 4. Prevalence of samples with high expression of Tier 2 targets. Heatmap spilt into panels A and B has the same layout as Figure 2; with the color scale represents the proportion of samples shown high expression (4th quartile) in a tumor type or in a normal tissue type. Expr: expression. BM: bone marrow. NA: not available. GTEx normal tissue: n=7,460; tumor samples: n=1,532. Supplementary references: 1, 2, 3, 4.

Supplementary Figure 5

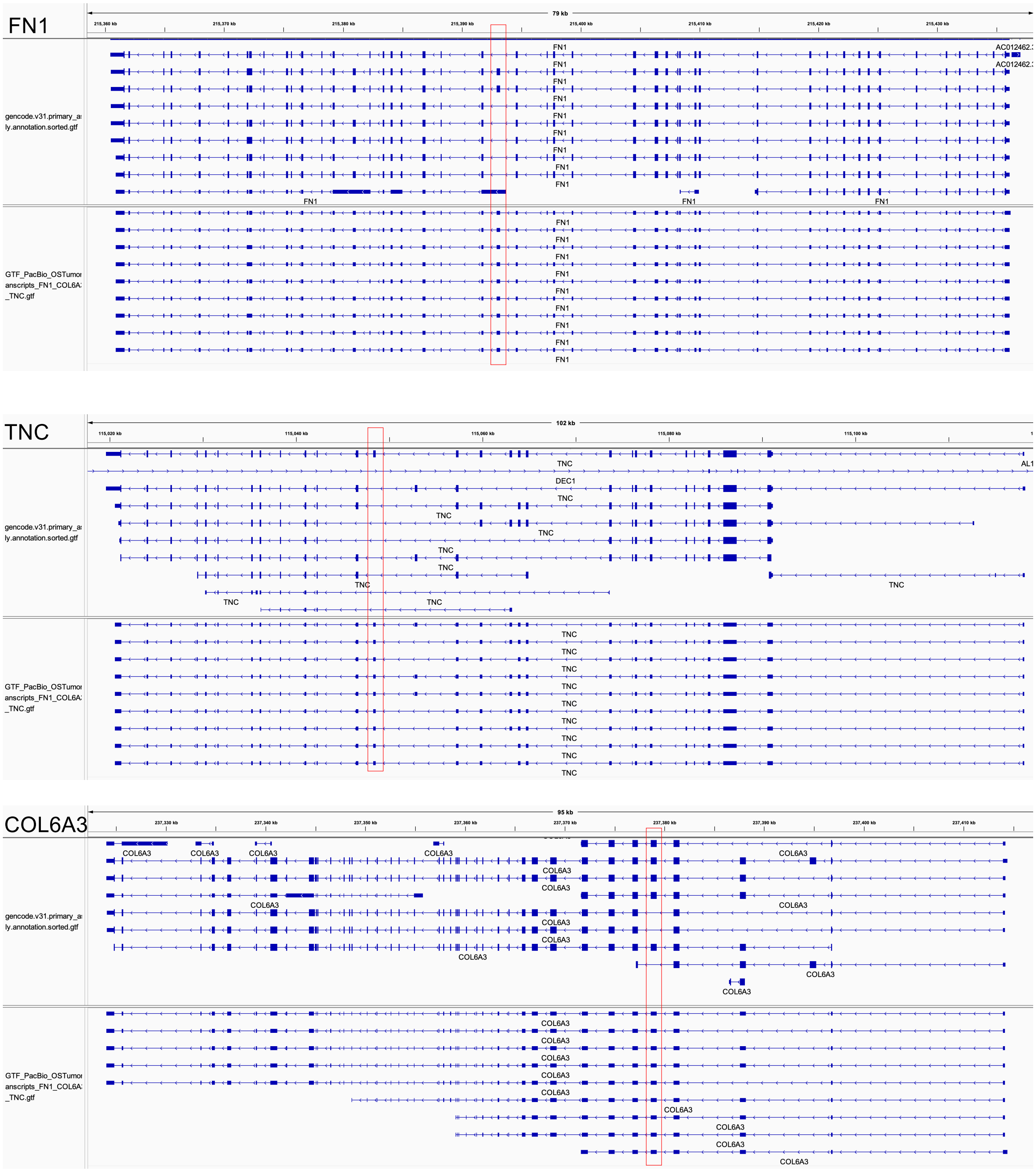

Supplementary Figure 5: Iso-seq full-length transcripts for three AS targets. FN1, COL6A3 and TNC are expressed at high levels in 83%, 34%, and 29% OS samples. For each gene. **Top panel:** annotated genecode transcripts. **Bottom panel:** 3-top transcript sequence that match the AS targets for each sample. The AS targets are highlighted with red boxes. Supplementary references: 5.

Supplementary Figure 6

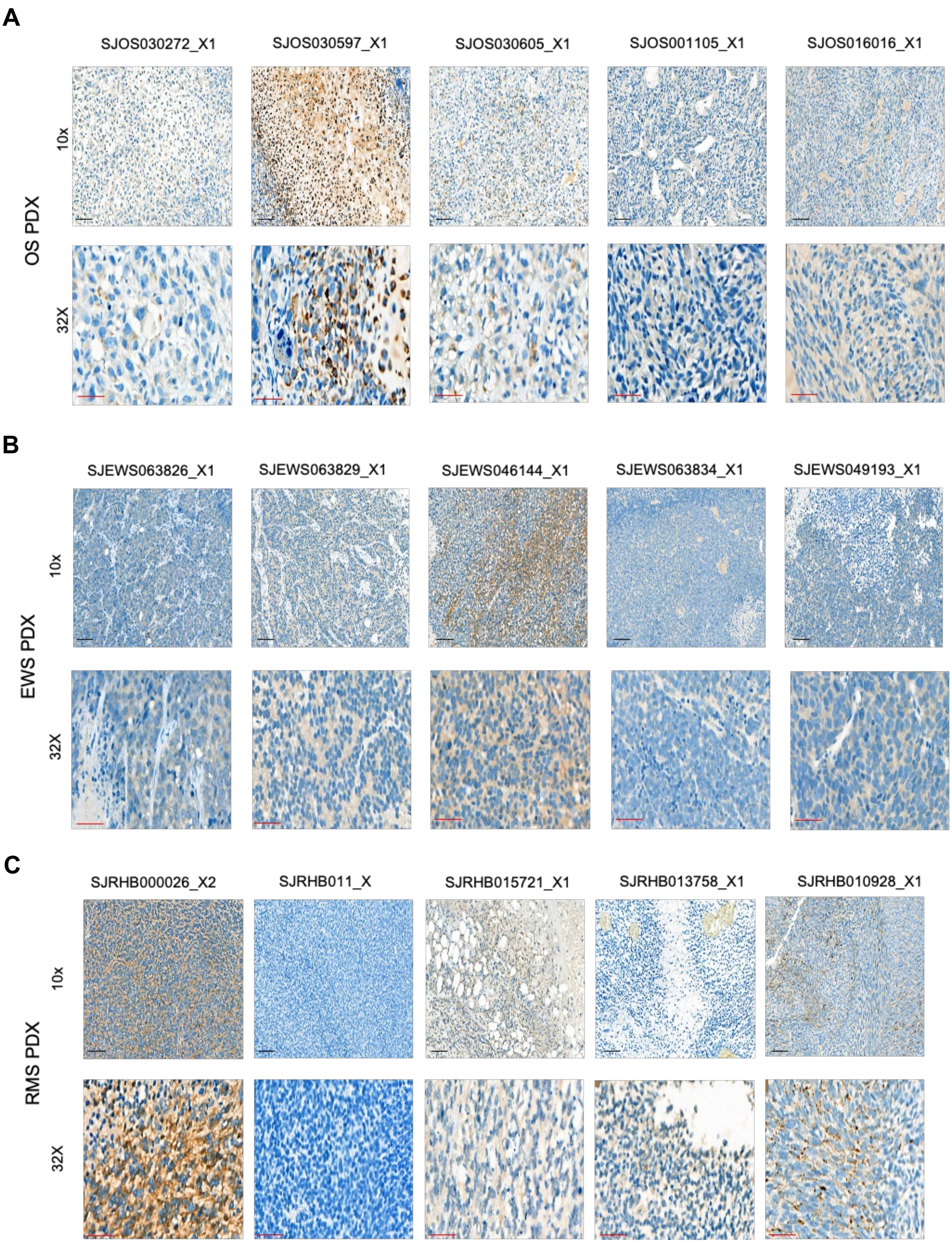

Supplementary Figure 6. Representative IHC images of COL11A1 expression in OS, EWS, and RMS PDX samples. The COL11A1-specific mAb 1e8.33 was used to detect COL11A1 by IHC. (**A**) OS, (**B**) EWS, (**C**) RMS. For each panel: top row: 10x magnification; scale bar, 100 µm (black). Bottom row: 32x magnification; scale bar, 50 µm (red). Each image is representative of one PDX tumor (OS: n=5, EWS: n=5, RMS: n=5).

Supplementary Figure 7

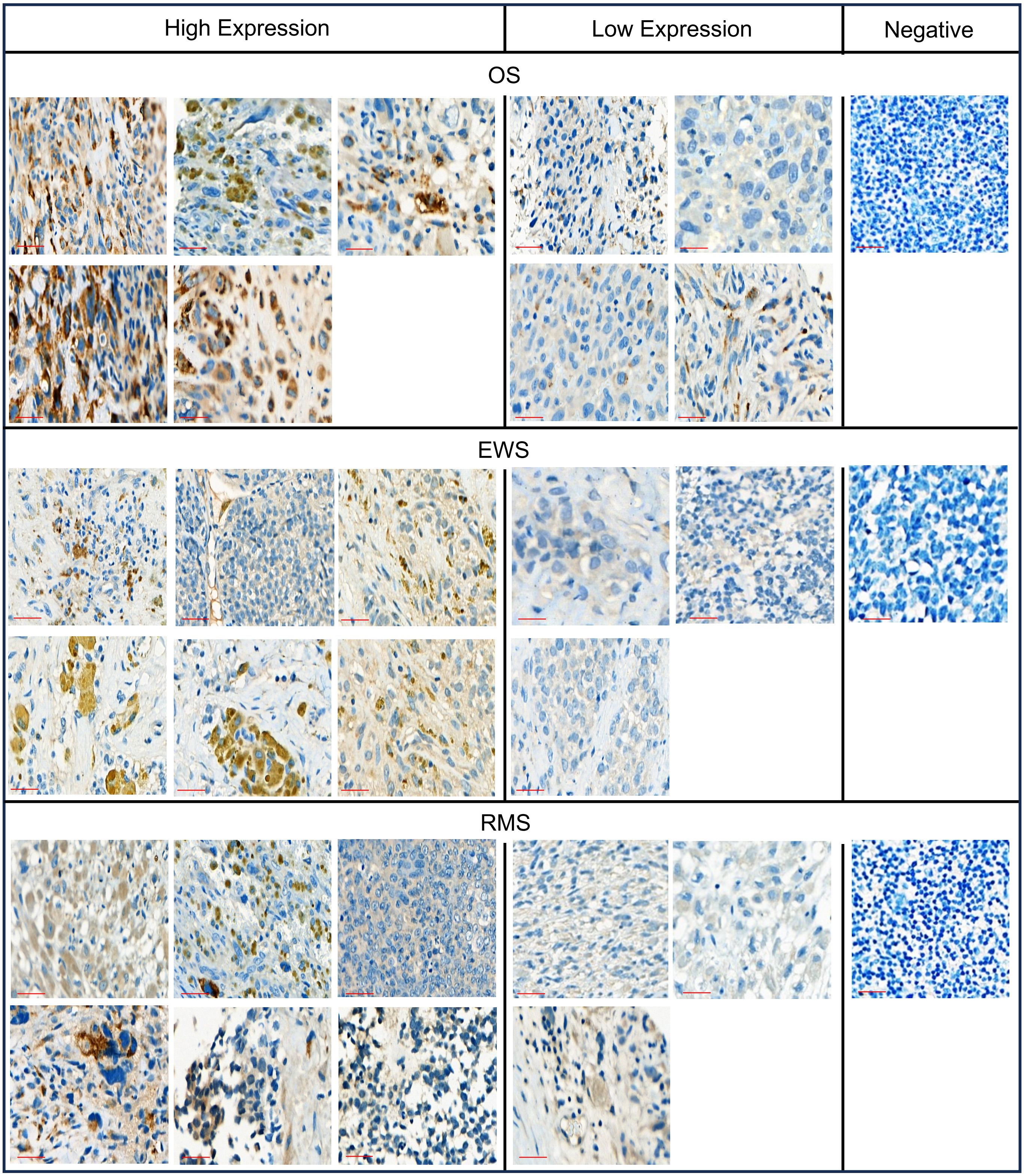

Supplementary Figure 7. Representative IHC images of COL11A1 expression in primary OS, EWS, and RMS samples. The COL11A1-specific mAb 1e8.33 was used to detect COL11A1 by IHC; 18 OS, 11 EWS, and 37 RMS were analyzed. 32x magnification; scale bar, 50 µm (red). Each image is representative of one primary tumor (OS: n=10, EWS: n=10, RMS: n=10).

Supplementary Figure 8

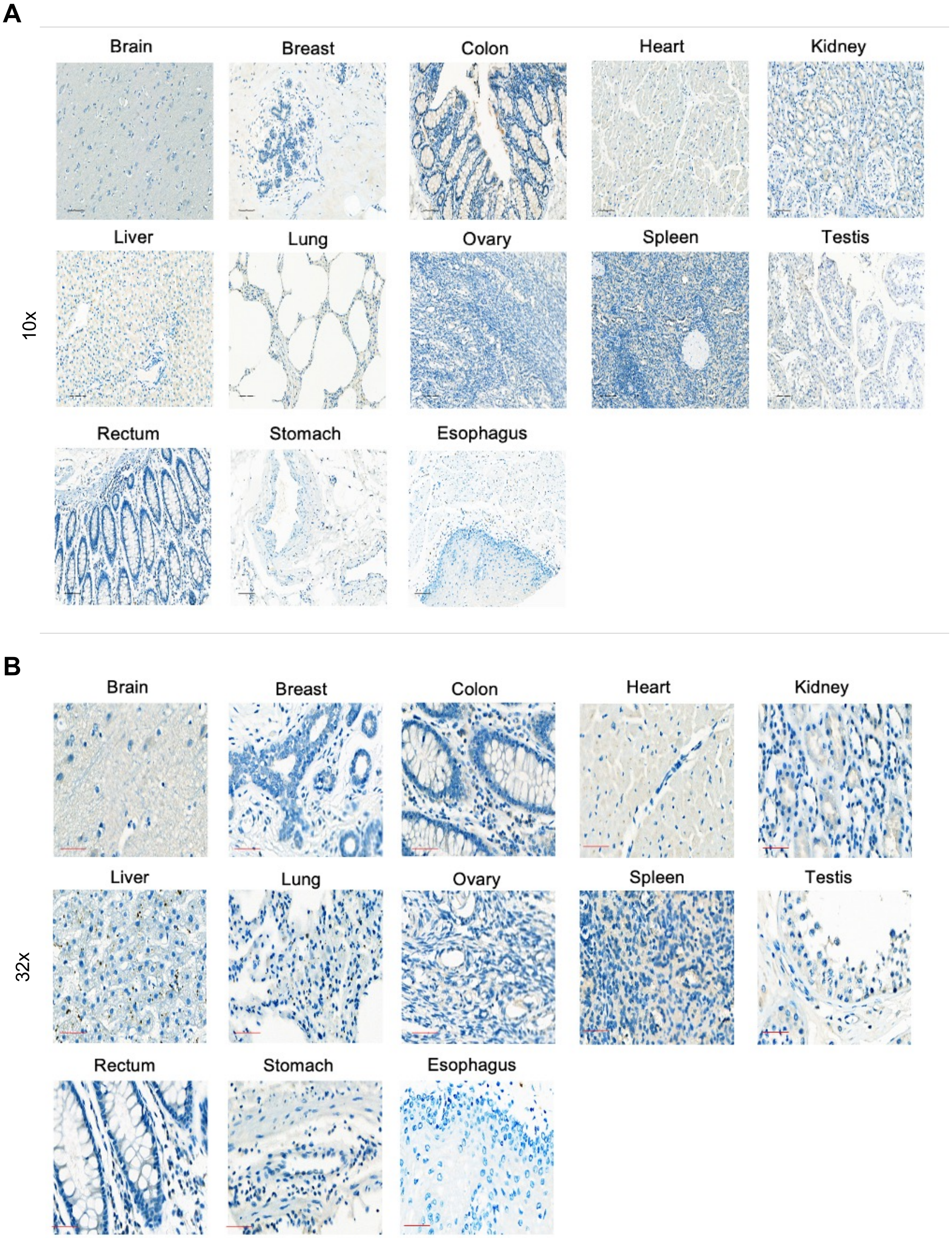

Supplementary Figure 8. Representative IHC images of COL11A1 expression in normal healthy tissues samples. The COL11A1-specific mAb 1e8.33 was used to detect COL11A1 by IHC. (**A**) Images, 10x magnification; scale bar, 100 μm (black). (**B**) Images, 32x magnification; scale bar, 50 μm (red). Each image is a representative of one normal organ. The TMA contained six cores per normal organ.

Supplementary Figure 9

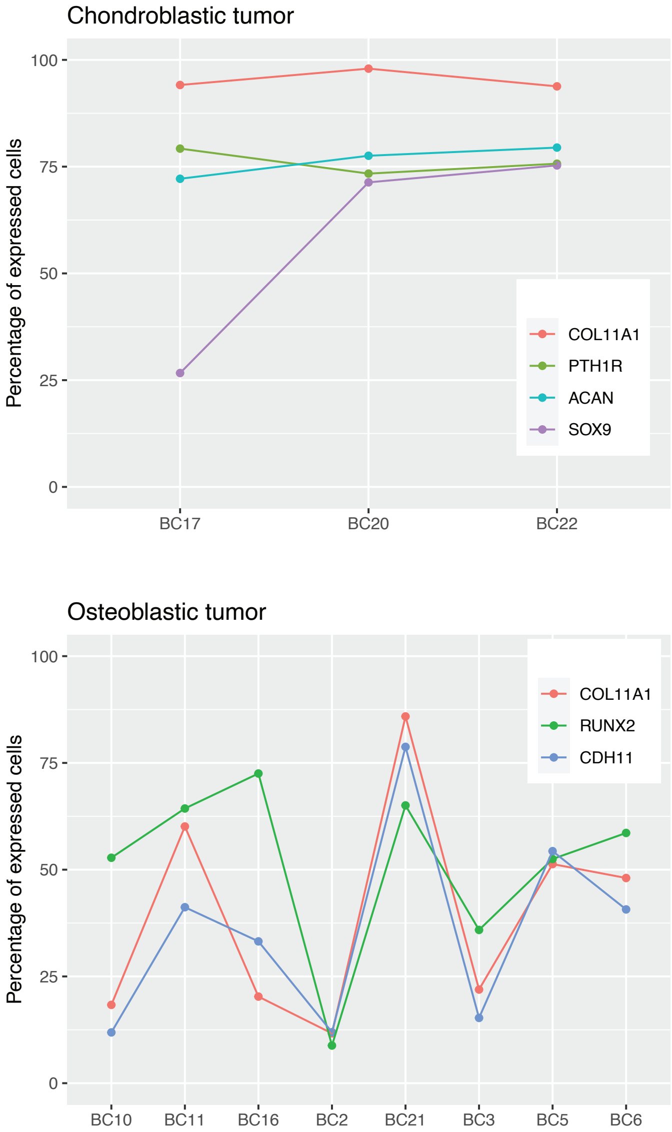

Supplementary Figure 9. Expression prevalence of *COL11A1* in osteosarcoma tumor cells using single-cell RNA-seq data from Zhou *et al* (PMID 33303760). The tumor cells were identified based on marker genes expressed in chondroblast cells and osteoblast cells for 3 chondroblastic OS tumors, and 8 osteoblastic OS tumors, respectively, as documented by Zhou et al. The marker genes for chondroblastic OS are PTH1R, ACAN, SOX9 while those for osteoblastic OS are RUNX2 and CDH11. *COL11A1* expression at the single cell level is comparable to marker genes in all samples except for BC16. Supplementary reference: 6.

Supplementary Figure 10

A

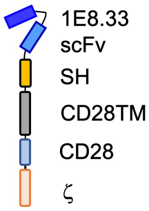

B

COL11A1-CAR.2A.tCD19: 1E8.33 scFv.SH.CD28TM.CD28.zeta.2A.tCD19
RED: Leader Sequence
Blue: 1E8.33 scFv
ORANGE: Short Hinge (SH)
BROWN: CD28 Transmembrane Domain (TM)
GREY: CD28.zeta
GREEN:2A
PINK: tCD19

Amino Acid Sequence:
MDWIWRILFLVGAATGAHSEVQLQESGPELVKTGSSVKISCKASGYSFTGYYMHWVKQSQGKSLEWIGYINCYNG
ATSYNRNFKGKATFTVDTSSSTAYMQFNSLTSGDSAVYYCAIWDYEFHVMDYWGQRTSLTVGGGGSGGGGSGG
GGSENVLTQSPAIMSASLGEKVTMSCRASSSVNYMYWYQQKSDASPKLWIYYTSNLAPGVPARFSGSGSGNSYS
LTISSMEGEDADTYYCQQFTSSPWTFGGGTKLEIKDLEPKSCDKTHTCPPCPDPKFWVLVVVGGVLACYSLLVTVA
FIIFWVRSKRSRLLHSDYMNMTPRRPGPTRKHYQPYAPPRDFAAYRSRVKFSRSADAPAYQQGQNQLYNELNLGR
REEYDVLDKRRGRDPEMGGKPRRKNPQEGLYNELQKDKMAEAYSEIGMKGERRRGKGHDGLYQGLSTATKDTY
DALHMQALPPRASRAEGRGSLLTCGDVEENPGPMPPPRLLFFLLFLTPMEVRPEEPLVVKVEEGDNAVLQCLKGT
SDGPTQQLTWSRESPLKPFLKLSLGLPGLGIHMRPLAIWLFIFNVSQQMGGFYLCQPGPPSEKAWQPGWTVNVEG
SGELFRWNVSDLGGLGCGLKNRSSEGPSSPSGKLMSPKLYVWAKDRPEIWEGEPPCLPPRDSLNQSLSQDLTMA
PGSTLWLSCGVPPDSVSRGPLSWTHVHPKGPKSLLSLELKDDRPARDMWVMETGLLLPRATAQDAGKYYCHRG
NLTMSFHLEITARPVLWHWLLRTGGWKVSAVTLAYLIFCLCSLVGILHLQRALVLRRKRKRMTDPTRRF

C **D**

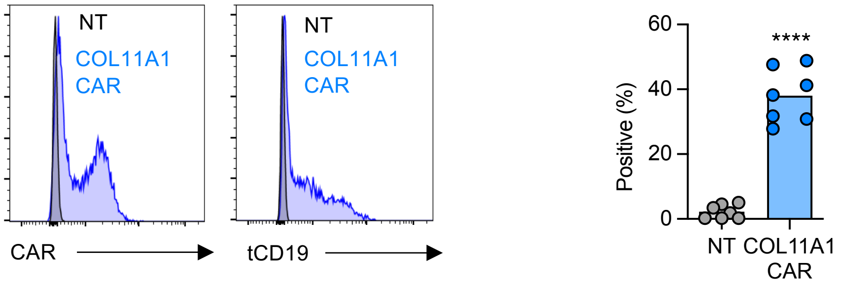

Supplementary Figure 10. Generation of COL11A1-CAR T cells. (**A**) Structure of COL11A1-CAR: 1E8.33 scFv, M13 short hinge, CD28 transmembrane and costimulatory domain, and a CD3 zeta signaling domain. (**B**) Amino acid sequence of gene encoding COL11A1-CAR-2A-tCD19. (**C**) Representative FACS plots of transduced T cells. Left panel: detection of CAR with anti-mouse IgG (Fab')2 fragment; Right panel: detection of tCD19 with anti-CD19. (**D**) Summary data for tCD19 expression, n=7, t-test, ****p<0.0001.

Supplementary Figure 11

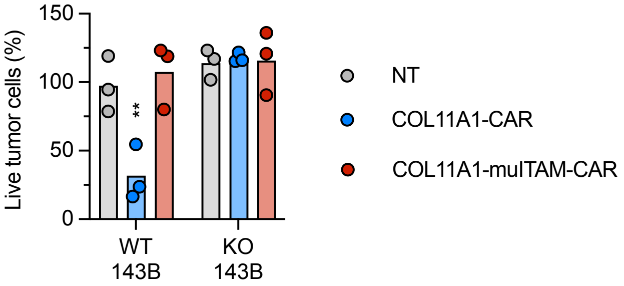

Supplementary Figure 11: Recognition of target cells by COL11A1-CAR T cells depends on the expression of COL11A1 and functional COL11A1-CARs. NT, COL11A1-CAR T-cells, and COL11A1-muITAM-CAR T cells were cocultured with indicated tumor cells at a 4:1 E:T ratio. After 72 hours live tumor cells were determined by MTS assay (n=3 biologically independent donors , two-way ANOVA, **p=0.0025).

Supplementary Figure 12

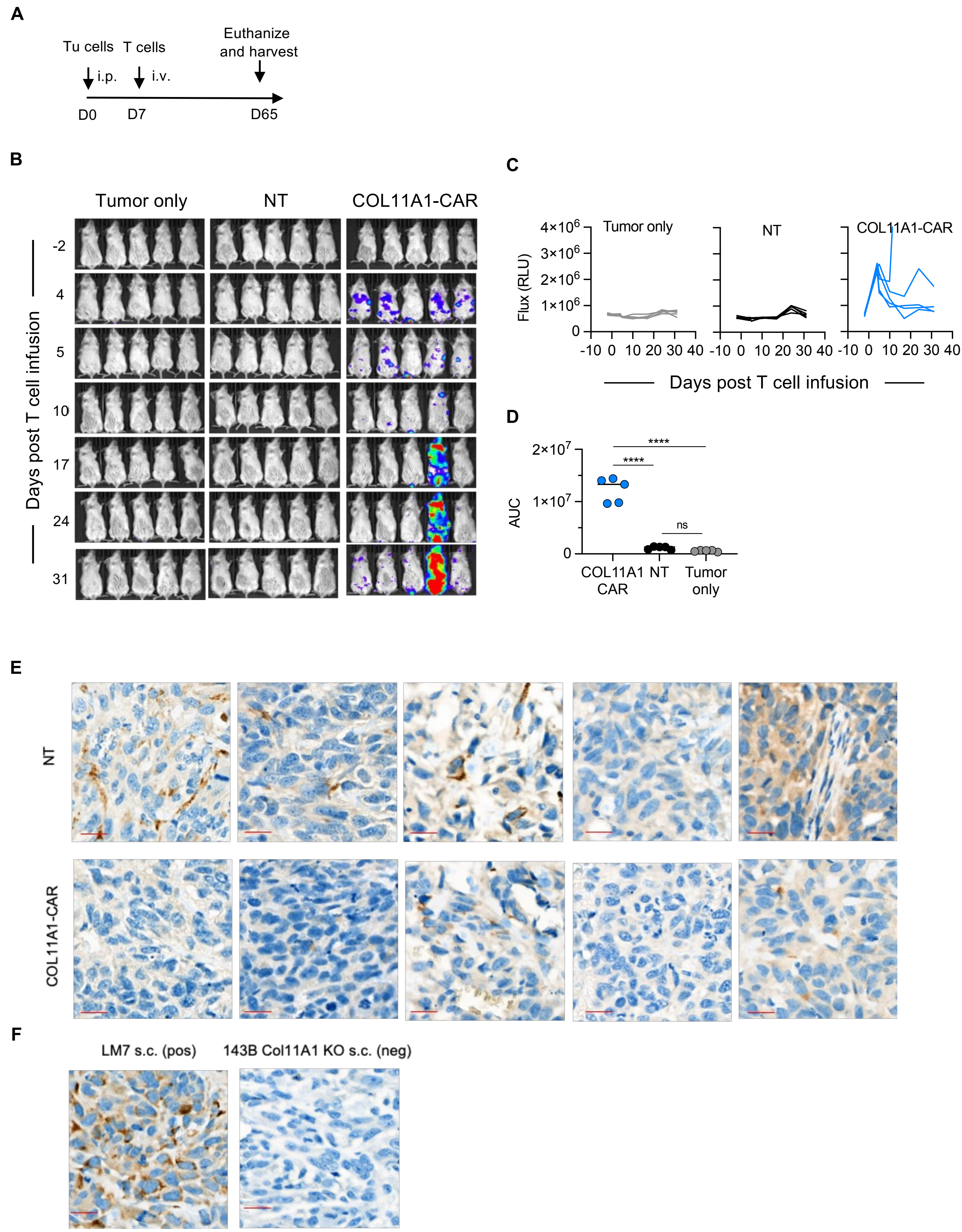

Supplementary Figure 12. Transient *in vivo* expansion of COL11A1-CAR T cells and recurrence of tumors with decreased expression of COL11A1. (**A**) Schematic of animal experiment. Day 0: i.p. injection of $1x10^6$ LM7 cells; D7: i.v. injection of $3x10^6$ NT or CAR T cells genetically modified to express eGFP.ffLuc (n=5 mice per group). (**B**) All bioluminescence images are shown (n=5 mice per group). (**C**) Quantitative bioluminescence data. (**D**) Area under the curve analysis for the first 10 days post T cell infusion, two-way ANOVA, ****p<0.0001, ns: not significant. (**E**) Representative IHC images for Col11A1 of tumors harvested after 65 days post T cell injection (n=5, 1 image per tumor); 32x magnification, scale bar, 50 µm (red). (**F**) Representative IHC images for Col11A1 expression of positive control tumor (LM7; subcutaneous tumor: s.c.), and negative control tumor (143B-COL11A1-KO; s.c.) used for all IHC experiments throughout the manuscript. 32x magnification; scale bar, 50 µm (red).

Supplementary Figure 13

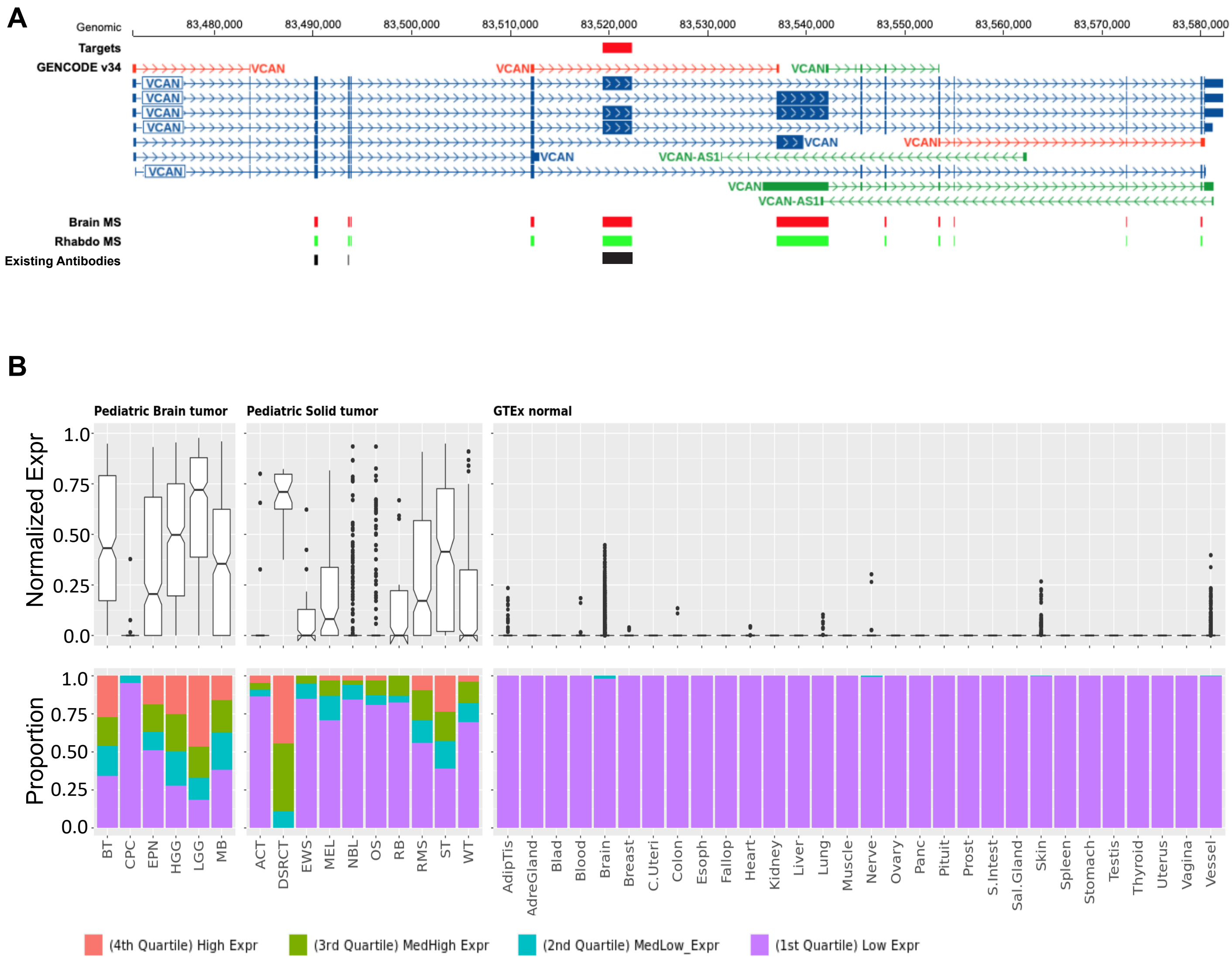

Supplementary Figure 13. Gene expression pattern of a VCAN AS exon target across tumor types and normal tissues. (**A**) Gene structure of VCAN. The AS exon identified as Tier1 target is shown as a red rectangle. MS: mass spectrometry. (**B**) Normalized expression shown high expression in tumor (n=1,532) and no expression in normal (n=7,460) tissues. Boxplot showing the exon expression in rank normalized percentile in the panel above and bar plot showing the quartile distribution across solid and brain tumors in the panel below. In the notched box plot, the lower, middle and upper hinges of the box plots correspond to the 25th, 50th and 75th percentiles, respectively. The notch displays the 95% confidence interval of the median. The upper whisker of the box plot extends from the upper hinge to the largest value no further than 1.5 × IQR from the upper hinge. IQR, interquartile range or distance between 25th and 75th percentiles. The lower whisker extends from the lower hinge to the smallest value at most 1.5 × IQR from the lower hinge. Data beyond the end of the whiskers are outlier points. Both (**A**) and (**B**) were generated from CSEMiner data portal. Supplementary references: 1, 2, 3, 4, 7.

Supplementary Figure 14

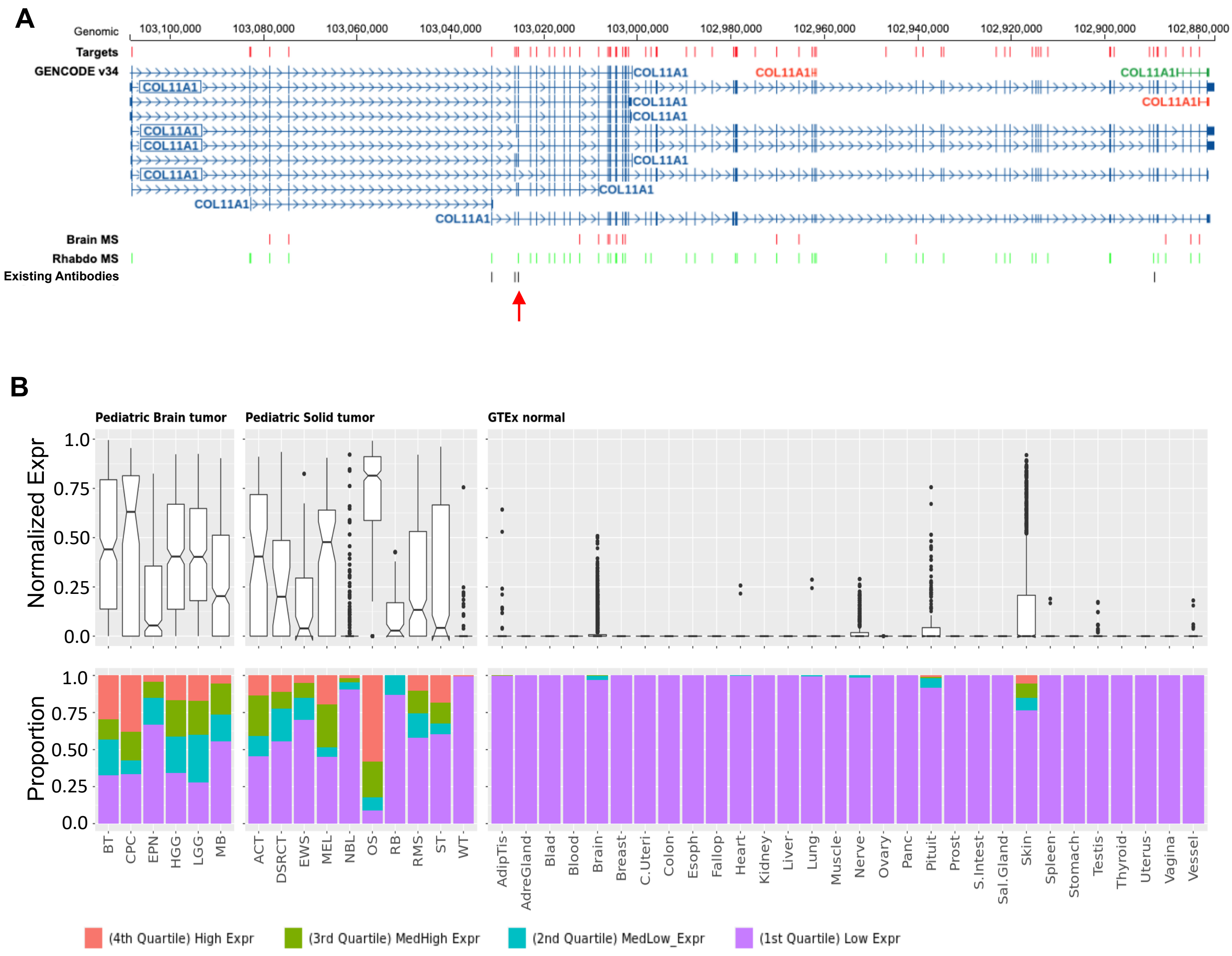

Supplementary Figure 14. Expression pattern of COL11A1 in tumor types and normal tissues. COL11A1 is a gene-level target and is transcribed in reverse orientation of the reference human genome. (**A**) Gene structure of COL11A1. The exon selected for gene expression display in panel B is marked by a red arrow. MS: mass spectrometry. (**B**) Normalized expression shown high expression in tumor (n=1,532) and no expression in normal (n=7,460) tissues. Boxplot showing the exon expression in rank normalized percentile in the panel above and bar plot showing the quartile distribution across solid and brain tumors in the panel below. In the notched box plot, the lower, middle and upper hinges of the box plots correspond to the 25th, 50th and 75th percentiles, respectively. The notch displays the 95% confidence interval of the median. The upper whisker of the box plot extends from the upper hinge to the largest value no further than 1.5 × IQR from the upper hinge. IQR, interquartile range or distance between 25th and 75th percentiles. The lower whisker extends from the lower hinge to the smallest value at most 1.5 × IQR from the lower hinge. Data beyond the end of the whiskers are outlier points. Both panels were generated from CSEMiner web portal. Both (**A**) and (**B**) were generated from CSEMiner data portal. Supplementary references: 1, 2, 3, 4, 7.

Supplementary Figure 15

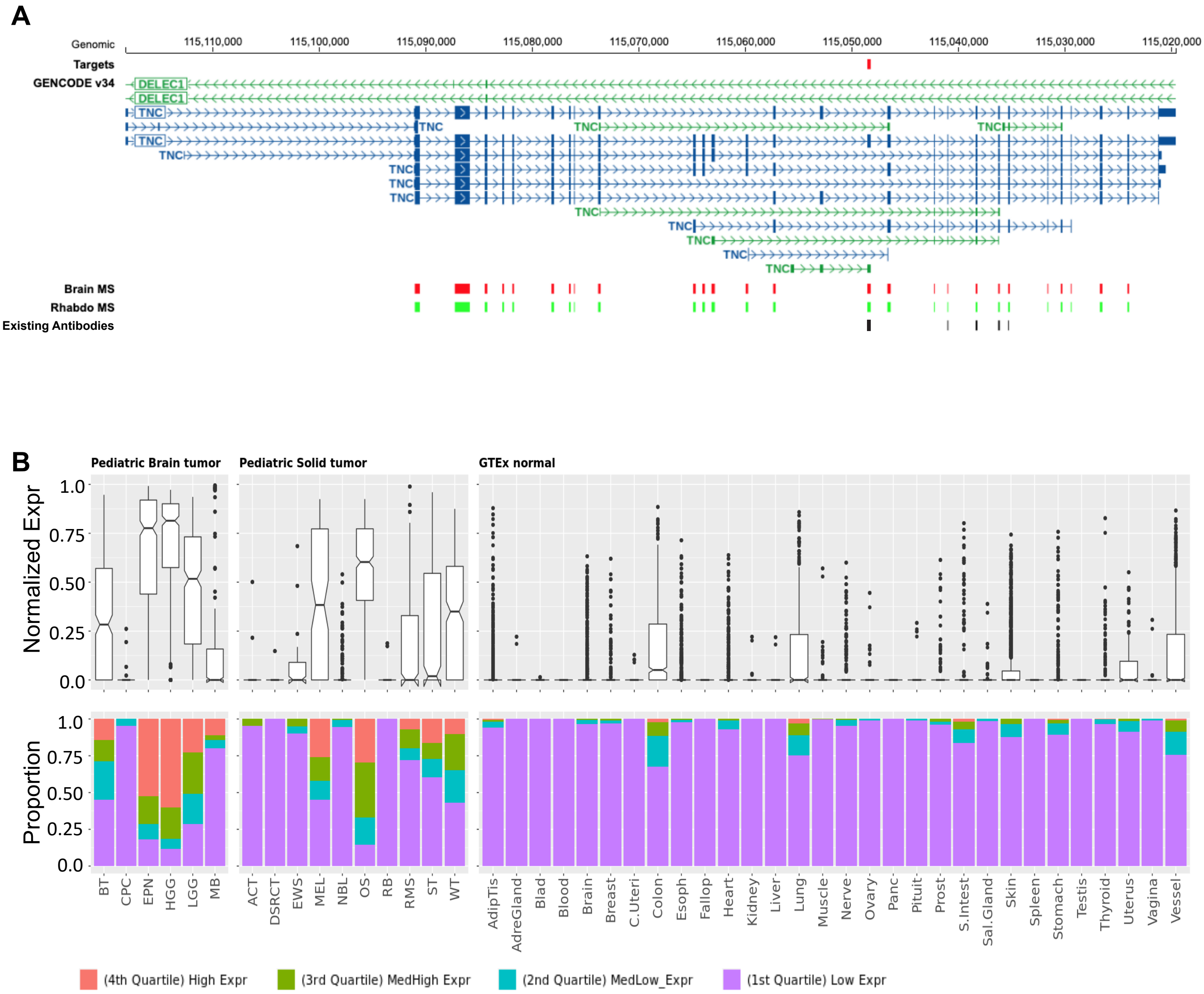

Supplementary Figure 15. Gene expression pattern of a TNC AS exon target across tumor types and normal tissues. (**A**) Gene structure of TNC that is transcribed in reverse orientation of the reference human genome. The AS exon identified as Tier1 target is shown as a red rectangle. MS: mass spectrometry. (**B**) Normalized expression shown high expression in tumor and (n=1,532) no expression in normal (n=7,460) tissues. Boxplot showing the exon expression in rank normalized percentile in the panel above and bar plot showing the quartile distribution across solid and brain tumors in the panel below. In the notched box plot, the lower, middle and upper hinges of the box plots correspond to the 25th, 50th and 75th percentiles, respectively. The notch displays the 95% confidence interval of the median. The upper whisker of the box plot extends from the upper hinge to the largest value no further than 1.5 × IQR from the upper hinge. IQR, interquartile range or distance between 25th and 75th percentiles. The lower whisker extends from the lower hinge to the smallest value at most 1.5 × IQR from the lower hinge. Data beyond the end of the whiskers are outlier points. Both (**A**) and (**B**) were generated from CSEMiner data portal. Supplementary references: 1, 2, 3, 4, 7.

Supplementary Figure 16

A

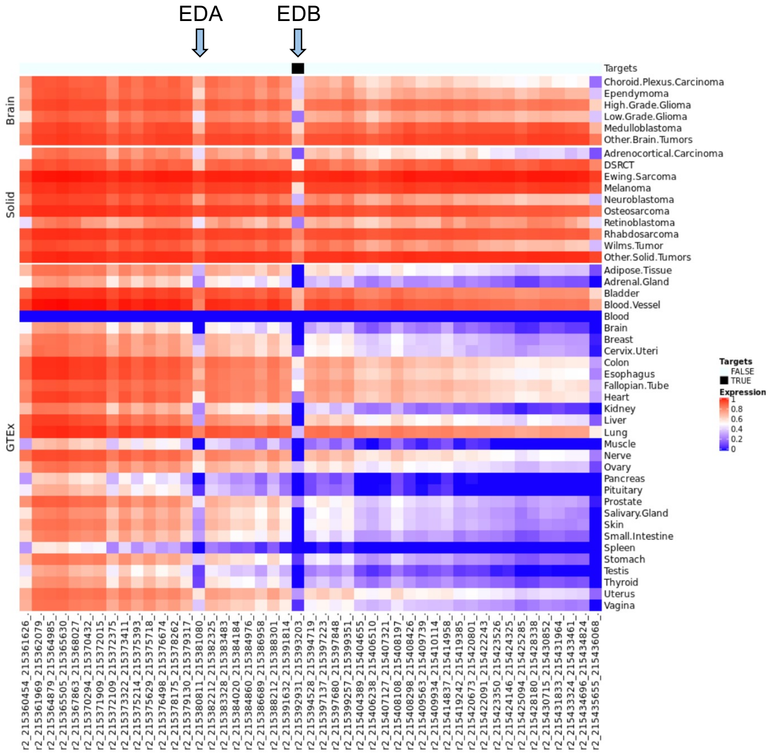

B

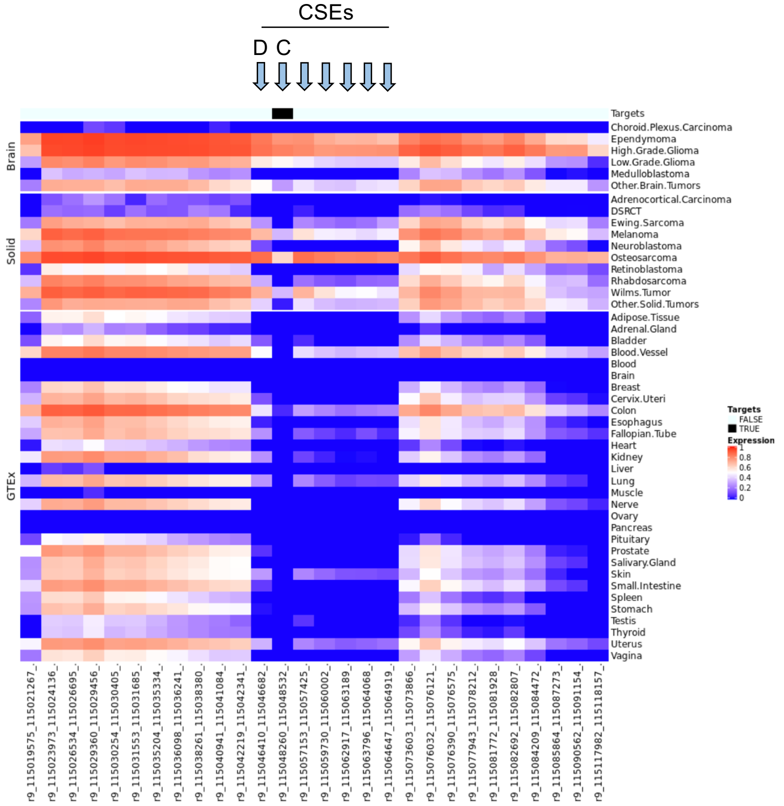

Supplementary Figure 16. CSE targets in FN1 and TNC. Heatmaps showing exon expression across normal tissue types (n=7,460) for (**A**) **FN1** and (**B**) TNC. (**A**) Exclusion of exon that encodes extra domain A (EDA) due to expression in normal tissues. (**B**) Seven CSEs were identified in TNC, including the CSEs encoding the C and D domain of TNC, respectively. Supplementary references: 1, 2, 3, 4.

Supplementary Figure 17

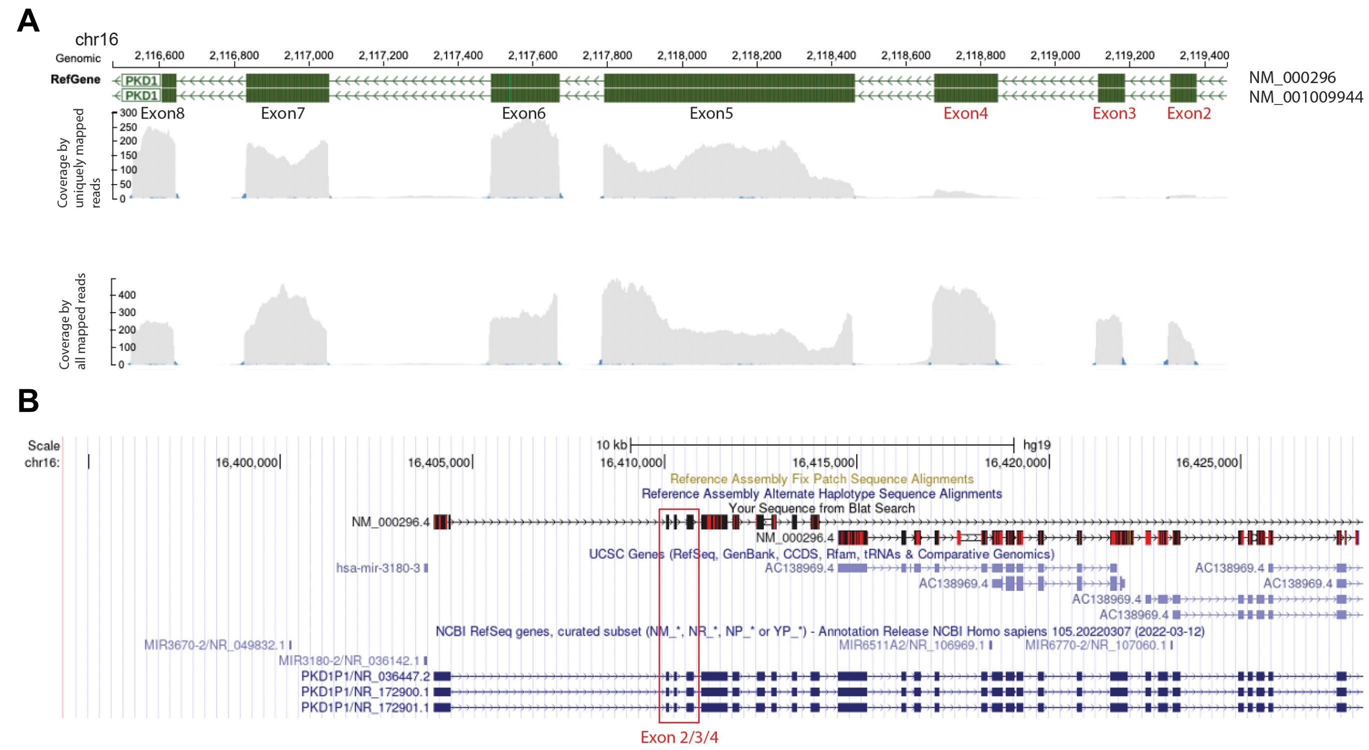

Supplementary Figure 17. PKD1 exon expression quantification affected by uniqueness of RNA-seq mapping. (**A**) Coverage of RNA-seq of a GTEx brain cerebellum sample (SRR1307770) using the uniquely mapped reads (MAPQ ≥10, used as a default by htseq, top panel) and all mapped reads (MAPQ ≥0, bottom panel). Exons 2-4 labeled in red text shown no coverage using uniquely mapped reads (top) in contrast to comparable coverage using all reads (bottom panel). (**B**) Blat search of PKD1 mRNA shown that exons 2-4 of PDK1 share 100% sequence identity to the three exons in PKD1P1, a known pseudo gene located 14Mb downstream. This explains the non-unique mapping of these exons in RNA-seq data. Supplementary reference: 1.

Supplementary Figure 18

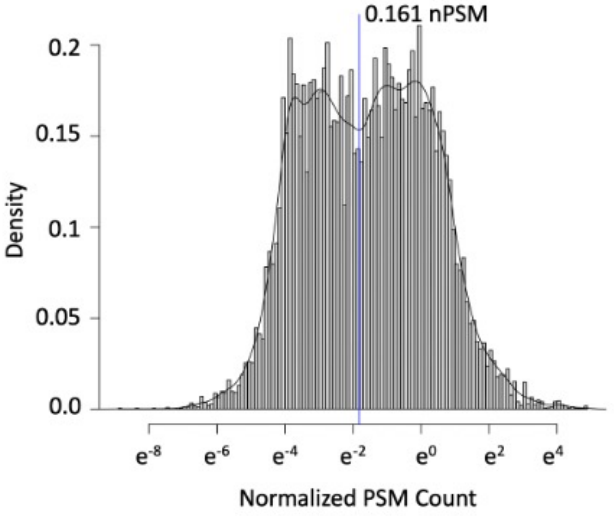

Supplementary Figure 18. Normalized PSM distribution in GTEx. A cutoff was determined based on the bimodal distribution was used to define highly expressed peptides at 0.161. Supplementary reference: 8.

Supplementary Figure 19

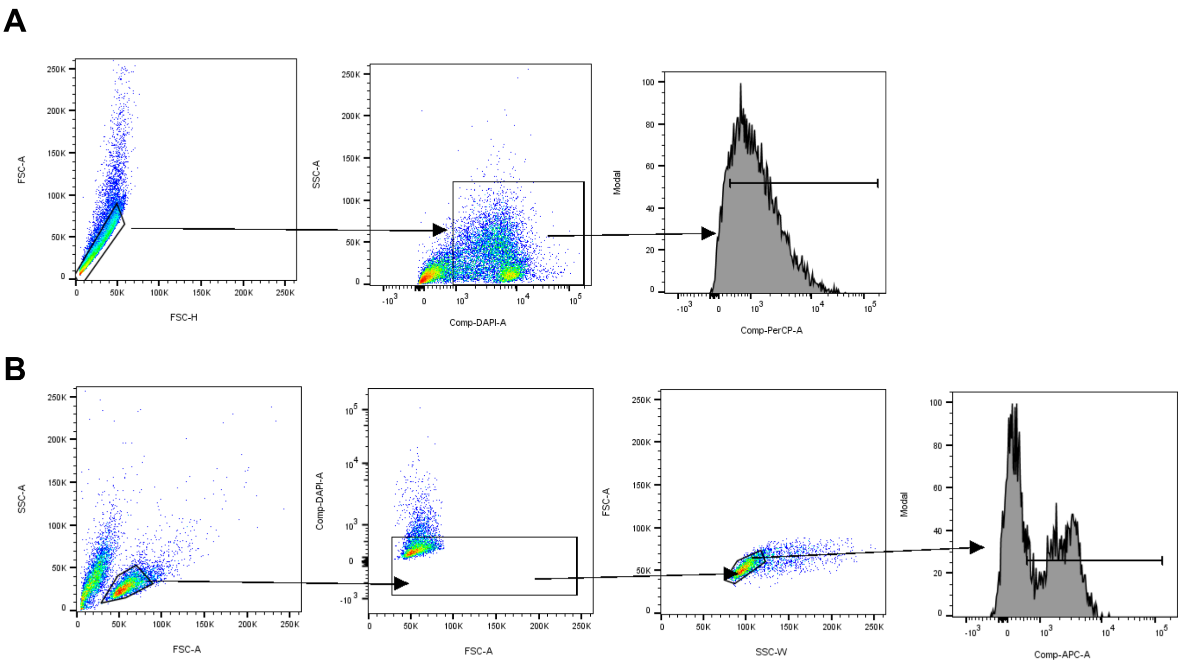

Supplementary Figure 19. Flow gating strategies. (**A**) Gating strategies for PDX samples: Singlet-live-gate on positive based on negative and positive controls. (**B**) Gating strategy for CART transduction: Lymphocyte-Live-Singlet-positive based on negative and positive controls.

SUPPLEMENTARY REFERENCES

	Name	URL	Searchable Accession
1	GTEx RNAseq	https://www.ncbi.nlm.nih.gov/projects/gap/cgi-bin/study.cgi?study_id=phs000424.v8.p2	
2	TARGET RNAseq	https://www.ncbi.nlm.nih.gov/projects/gap/cgi-bin/study.cgi?study_id=phs000218.v1.p1	
3	Clinical RNAseq	https://platform.stjude.cloud/data/cohorts/pediatric-cancer	SJC-DS-1003, SJC-DS-1004, SJC-DS-1007
4	PCGP RNAseq	https://platform.stjude.cloud/data/cohorts/pediatric-cancer	SJC-DS-1001
5	Long-read GTF File	https://zenodo.org/records/10844897	The iso-seq read data can be accessed in the European Genome-phenome Archive (EGA): Accession EGAS00001007766
6	Single cell analysis of osteosarcoma tissues	https://www.ncbi.nlm.nih.gov/geo/query/acc.cgi?acc=GSE152048	
7	Pediatric Mass Spectrometry	https://pdc.cancer.gov/pdc/study/PDC000180	
8	GTEx Mass Spectrometry	https://proteomecentral.proteomexchange.org/cgi/GetDataset?ID=PXD016999	
