# Supplementary material for: Discovery of immunotherapy targets for pediatric solid and brain tumors by exon-level expression
Source: Nat Commun. 2024 May 3;15:3732. doi: 10.1038/s41467-024-47649-y (PMC11068777; doi:10.1038/s41467-024-47649-y)
Supplement: Supplementary file 3 — Description of Additional Supplementary Files [file 41467_2024_47649_MOESM3_ESM.pdf]

SUPPLEMENTARY DATA LEGENDS

Supplementary Data 1: Identified Exon Candidates.
The **first tab** lists the 157 identified Tier 1 and Tier 2 exon candidates.
The **second tab** lists all exons upregulated in at least one tumor type with less than 5 high normal tissue expression and restricted to surfaceome and matrisome genes.
The **third tab** provides information on the annotation:
Oncofetal Annotation: GeneCard text mining of oncofetal gene list
ClusterOfDifferentiation*: HGNC annotation of the CD gene list. https://www.genenames.org/cgi-*bin/genegroup/download?id=471&type=node
Cell Surface Protein Atlas: mass-spectrometry-derived cell surface protein atlas. [Bausch-Fluck et al. PMID: 2589452],
Cell_differentiation: MsigDB v7.2 cell differentiation markers gene family
Compartment_CellSurface: Cell Surface genes derived from the COMPARTMENT database integrated channels accessed 2020/11/20
Compartment_ECM: Extracellular matrix genes derived from the COMPARTMENT database integrated channels accessed 2020/11/20", "AMIGO_ECM: AMIGO ECM annotation (http://amigo.geneontology.org/amigo/term/GO:0031012; accessed 20190226; PMID: 19033274)
MatrixDB: Matrisome CORE protein from MatrixDB
HPA_Predicted_Secreted: The human protein atlas (HPA) was accessed 2020-11-20. [Uhlen et al. PMID: 25613900] Proteins predicted to be secreted and compiled by HPA
HPA_MembraneBound: The human protein atlas (HPA)was accessed 2020-11-20. Proteins were predicted to be secreted
MGI_ExtracellularRegion: MGI extracellular region GO:0005576 accessed 3/3/2019
MGI_ExtrinsicCase: MGI extrinsic component of membrane GO:0019898 accessed 3/3/2019
MGI_ECM: MGI extracellular matrix GO:0031012 accessed 3/3/2019
MGI_ExternalSidePM: MGI external side of plasma membrane GO:0009897 accessed 3/3/2019
TRUE indicate the gene is present in the gene list.
The fourth tab provides definitions.

Supplementary Data 2: Genomic Variants at +/-10bp of donor/splice sites of AS targets. The genomic variants at +/-10bp of donor/splice sites of two alternative spliced (AS) targets (FN1 AS exon at chr2_215392931_215393203_-; VCAN AS exon at chr5_83519349_83522309_+) are provided. The used method is described in the method section of the manuscript.

Supplementary Data 3: PDX Sample Information. Detailed information on the patient derived xenograft (PDX) samples used in this study are provided. The link to individual PDX information in the St. Jude Childhood Solid Tumor Network (CSTN) Data Portal is provided.

Supplementary Data 4: RNA-seq Sample Accession. All RNA-seq sample accession numbers for the manuscript are provided.
