# Supplementary material for: Discovery of immunotherapy targets for pediatric solid and brain tumors by exon-level expression
Source: Nat Commun. 2024 May 3;15:3732. doi: 10.1038/s41467-024-47649-y (PMC11068777; doi:10.1038/s41467-024-47649-y)
Supplement: Supplementary file 8 — Reporting Summary [file 41467_2024_47649_MOESM8_ESM.pdf]

nature portfolio

Corresponding author(s): Jinghui Zhang and Stephen Gottschalk

Last updated by author(s): Apr 3, 2024

Reporting Summary

Nature Portfolio wishes to improve the reproducibility of the work that we publish. This form provides structure for consistency and transparency in reporting. For further information on Nature Portfolio policies, see our Editorial Policies and the Editorial Policy Checklist.

Statistics

For all statistical analyses, confirm that the following items are present in the figure legend, table legend, main text, or Methods section.

n/a	Confirmed	
☐	☒	The exact sample size (*n*) for each experimental group/condition, given as a discrete number and unit of measurement
☐	☒	A statement on whether measurements were taken from distinct samples or whether the same sample was measured repeatedly
☐	☒	The statistical test(s) used AND whether they are one- or two-sided<br>*Only common tests should be described solely by name; describe more complex techniques in the Methods section.*
☒	☐	A description of all covariates tested
☐	☒	A description of any assumptions or corrections, such as tests of normality and adjustment for multiple comparisons
☐	☒	A full description of the statistical parameters including central tendency (e.g. means) or other basic estimates (e.g. regression coefficient) AND variation (e.g. standard deviation) or associated estimates of uncertainty (e.g. confidence intervals)
☐	☒	For null hypothesis testing, the test statistic (e.g. *F*, *t*, *r*) with confidence intervals, effect sizes, degrees of freedom and *P* value noted<br>*Give P values as exact values whenever suitable.*
☒	☐	For Bayesian analysis, information on the choice of priors and Markov chain Monte Carlo settings
☐	☒	For hierarchical and complex designs, identification of the appropriate level for tests and full reporting of outcomes
☒	☐	Estimates of effect sizes (e.g. Cohen's *d*, Pearson's *r*), indicating how they were calculated

Our web collection on statistics for biologists contains articles on many of the points above.

Software and code

Policy information about availability of computer code

Data collection	Flow cytometry data was collected using BD FACSCanto and analyzed with FlowJo vl0.<br>Bioluminescent images were acquired with a XenogenVIS-200 imager.<br>Cytotoxicity and ELISA assays were measured with a Tecan Infinite M Nano absorbance plate reader.<br>RT-qPCR assays were measured QuantStudio 6 Flex System from Thermo Fisher.<br>For IHC samples: Orthotopic patient-derived xenograft samples, collected under the Molecular Analysis of Solid Tumors (MAST) protocol, were provided by the Childhood Solid Tumor Network (CSTN) collection at St. Jude. After St. Jude Institutional Review Board approval, deidentified archival formalin-fixed paraffin-embedded tissue blocks from clinical patient tumor samples were cut and H&E-stained sections were reviewed for correct diagnosis and tumor content by a pediatric pathologist (SCK). Matched unstained tumor sections were then stained.<br>Solid and brain pediatric tumor RNA-seq data were downloaded from the St. Jude Cloud [PMID 33408242] (https://platform.stjude.cloud/data/cohorts/pediatric-cancer) for St. Jude/Washington University Pediatric Cancer Genome Project (PCGP) and St. Jude's Clinical Genomics (ClinGen) program. NCI TARGET data were downloaded from dbGaP under accession phs000218. RNA-seq data from the normal tissues were generated by the Genotype-Tissue Expression (GTEx) consortium 42 and downloaded from the GTEx portal (http://gtexportal.org, release v7).<br>We retained targets encoding surfaceome or matrisome based on the following data sets:<br>The Cell Surface Protein Atlas, the MGI GO annotation, the human protein atlas, MatrixDB49, and the compartment database.
Data analysis	Flow Cytometry analysis was performed with FlowJo vl0 (BD Biosciences). Cytotoxicity and cytokine data was calculated on Excel (Microsoft) and graphed on Prism (Graphpad). Bioluminescent images were analyzed using Living Image (Perkin). All RNA-seq data were processed by a custom pipeline (WRAP, https://github.com/gatechatl/DRPPM_Example_Input_Output/tree/master/ WRAP:Wrapper-for-my-RNAseq-

Analysis-Pipeline). Data was analyzed using a Ficher's two side test for significance.

For manuscripts utilizing custom algorithms or software that are central to the research but not yet described in published literature, software must be made available to editors and reviewers. We strongly encourage code deposition in a community repository (e.g. GitHub). See the Nature Portfolio guidelines for submitting code & software for further information.

Data

Policy information about availability of data

All manuscripts must include a data availability statement. This statement should provide the following information, where applicable:

- Accession codes, unique identifiers, or web links for publicly available datasets
- A description of any restrictions on data availability
- For clinical datasets or third party data, please ensure that the statement adheres to our policy

The raw RNA-seq data for PCGP and St Jude ClinGen samples are available on St Jude Cloud Genomics Platform (https://platform.stjude.cloud/data/cohorts/pediatric-cancer) under the accessions SJC-DS-1001, SJC-DS-1003, SJC-DS-1004 and SJC-DS-1007. NCI TARGET data are available in dbGaP under accession phs000218. NCI TARGET data are available in dbGaP under accession phs000218 https://www.ncbi.nlm.nih.gov/projects/gap/cgi-bin/study.cgi?study_id=phs000218.v1.p1. The GTEx RNAseq data used in this study can be accessed through the dbGAP accession phs000424.v8.p2 https://www.ncbi.nlm.nih.gov/projects/gap/cgi-bin/study.cgi?study_id=phs000424.v8.p2. The Iso-seq data used for verifying alternative splicing of FN1, TNC, COL6A3 in osteosarcoma can be accessed in the European Genome-phenome Archive (EGA) under accession number EGAS00001007766. The GTEx proteomics data used in this study can be accessed through PXD016999 https://proteomecentral.proteomexchange.org/cgi/GetDataset?ID=PXD016999. The processed pediatric brain tumor proteomics data used in this study can be accessed through the NCI proteomics data commons https://pdc.cancer.gov/pdc/study/PDC000432. Other data is available on request. PDX IDs and their associated accessions can be found at Supplementary Table 3.

Research involving human participants, their data, or biological material

Policy information about studies with human participants or human data. See also policy information about sex, gender (identity/presentation), and sexual orientation and race, ethnicity and racism.

Reporting on sex and gender	The only human samples that were specifically collected for this study were blood samples to generate CAR T cells. These were collected from healthy adult donors on the PACT protocol (Pro00008053) at St. Jude. Since the samples were de-identified before processing in the laboratory, no information on sex and gender is available.
Reporting on race, ethnicity, or other socially relevant groupings	Only de-identified donors were used to generate CAR T cells; thus no information on race, ethnicity, or other socially relevant groupings is available.
Population characteristics	Only de-identified donors were used to generate CAR T cells; thus no information on race, ethnicity, or other socially relevant groupings is available. In addition, orthotopic patient-derived xenograft (PDX) samples, collected under the Molecular Analysis of Solid Tumors (MAST) protocol, were provided by the Childhood Solid Tumor Network (CSTN) collection at St. Jude for this study. These were selected based on the diagnosis (osteosarcoma, Ewing's sarcoma, rhabdomyosarcoma). PDX information, including age and gender, is provided in Supplementary Table 3.
Recruitment	Research participants will be recruited from volunteer participants through St Jude electronic postings (elevators, hallways, St. Jude Today), word-of-mouth, active recruitment by study-staff. All recruitment materials will be approved by the IRB before use. Advertisements will indicate the nature of the study and provide a phone number for further information. While 'self-selection bias' cannot be excluded, we do not believe that this has an impact on our study in which we generated genetically modified T cells from healthy donors.
Ethics oversight	Institutional Review Board at St. Jude Children's Research Hospital

Note that full information on the approval of the study protocol must also be provided in the manuscript.

Field-specific reporting

Please select the one below that is the best fit for your research. If you are not sure, read the appropriate sections before making your selection.

☒ Life sciences ☐ Behavioural & social sciences ☐ Ecological, evolutionary & environmental sciences

For a reference copy of the document with all sections, see nature.com/documents/nr-reporting-summary-flat.pdf

Life sciences study design

All studies must disclose on these points even when the disclosure is negative.

Sample size	The murine studies were performed with n=4-15 for each group; this is a statistically rigorous sample size to detect differences in treatment groups. Cytotoxicity assays were performed with n=3-4 donors and performed in triplicate. Confocal imaging studies were performed with one biological replicate and imaged at least 80 cells. Phosphoproteome analysis was performed with 2 biological replicates. For other experiments, sample size is described in the figure legends or in Methods. No sample-size calculation was performed to predetermine treatment groups; therefore, a minimum of 3 samples per group were used in order to perform statistical comparisons.
Data exclusions	No data were excluded.

Replication	In vivo studies were repeated at least twice in independent experiments with similar results. CAR T cell transductions were performed at least 3 independent times with separate retroviral supernatant preparations; these were then used for all subsequent assays. In vitro assays were performed with at least two biological replicates. For all experiments, attempts at replication were successful.
Randomization	Mice were consistently randomized when treating with immune cells all mice had similar tumor burdens between groups at day 7 (LM7, A673). No commonly misidentified cells lines were used in the study. Allocation of all samples were consistently randomized.
Blinding	Our measurements/analyses are not subjective. Thus, there is no requirement for blind experiments.

Reporting for specific materials, systems and methods

We require information from authors about some types of materials, experimental systems and methods used in many studies. Here, indicate whether each material, system or method listed is relevant to your study. If you are not sure if a list item applies to your research, read the appropriate section before selecting a response.

Materials & experimental systems

n/a	Involved in the study	
☐	☒	Antibodies
☐	☒	Eukaryotic cell lines
☒	☐	Palaeontology and archaeology
☐	☒	Animals and other organisms
☒	☐	Clinical data
☒	☐	Dual use research of concern
☒	☐	Plants

Methods

n/a	Involved in the study	
☒	☐	ChIP-seq
☐	☒	Flow cytometry
☒	☐	MRI-based neuroimaging

Antibodies

Antibodies used	Primary antibodies and probes with their dilutions: anti-F(ab')2-AF647 (109-606-006, Jackson Labs, Bar Harbor, ME, USA; 1:100), Anti-Human CD3e-AlexaFluor 647 (1:100) (Biolegend: #100209) , PanCollAl (Invitrogen, PAS-101300), VCAN (Novus NBP2-22408), and mAb anticol11A1 (Oncomatryx, High Concentration 2.3 mg/mL Rabbit monoclonal (Clone 1e8.33)) were used.<br>Antibodies conjugated using Lightning-Link" Labeling Kits (Novus Bio) according to the manufacturer's instructions. L19 MAb was synthesized with a hlgGl heavy chain and human kappa light chain (ThermoFisher Scientific) based on the L19 sequence of our CAR and a previous report in the literature.
Validation	All antibodies used in flow cytometry and immunofluorescence were titrated and validated. Antibodies used for flow cytometry were compared to untransduced controls. For surface antigens (VCAN, L19, CollAl), antibodies were compared to the listed negative and positive controls. (VCAN: neg, healthy fibroblast; pas, A673. EDB: neg, A549FN1-/-; pas, A549. CollAl: neg, healthy fibroblast; pos, LM7). Manufacturer validation statements can be found below for the corresponding antibodies. All antibodies are from commercially available sources (see above) and have been validated by the manufactures.<br>For IHC: 1e8.33 mAb (Oncomatryx) was validated using manufactures instructions along with negative control (Col11A1--/- A549) and positive controls (A673).

Eukaryotic cell lines

Policy information about cell lines and Sex and Gender in Research

Cell line source(s)	143b osteosarcoma, CCL-136 rhabdomyosarcoma, CRL-2061 rhabdomyosarcoma, and A673 Ewings sarcoma cell lines were obtained and grown as per American Type Culture Collection (ATCC, Manassas, VA, USA) instructions. LM7, a metastatic osteosarcoma cell line, was provided by Dr. Eugenie Kleinerman (MD Anderson Cancer Center, Houston, Texas, USA) in 2011. Primary fibroblast (Fib) cell lines from healthy donors were previously established (Blood (2003):101, 1905-1912).
Authentication	Cell lines were routinely validated using the ATCC STR Profiling Cell Authentication Service.
Mycoplasma contamination	All cell lines used were mycoplasma free as tested routinely using MycoAlert Mycoplasma Detection kit (Lonza, Walkersville, MD).
Commonly misidentified lines (See ICLAC register)	No commonly misidentified cell lines were used in the study.

Animals and other research organisms

Policy information about studies involving animals; ARRIVE guidelines recommended for reporting animal research, and Sex and Gender in Research

Laboratory animals	NOD-scid IL2Rgammanull mice (NSG) were obtained from breeding colonies maintained by the St. Jude Animal Resource Center. For all in vivo experiments, 7-8 weeks old mice were used. Mice allocated to different experimental groups were sex-, age-, and housingmatched.<br>Rodents are kept under barrier conditions in the ARC to keep them specific pathogen free. A clean-to-dirty traffic pattern is used in most corridors. In all corridors, employees enter a vestibule and apply applicable PPE before entering the corridor and animal rooms to work. All cages, food, bedding and supplies are sterilized in bulk autoclaves. Rodents are maintained in microisolation caging and cage changes are performed under a change station or Class 2A biological safety cabinet. Differential airflow is used as a preventative measure in cross contamination.<br>Microisolation cages (cages with filter tops or cages that fit into special ventilated racks) are used to house rodents within the facility. During “daylight hours” animal rooms are maintained on the low-intensity white light setting. Evening hours activate a "red light" setting. The lights in most animal rooms and corridors of the ARC are on an automated 12 hour on, 12 hour off light cycle. Other light cycles can be set if necessary for research objectives.<br>Each animal room and cubicle room in the ARC has a separate thermostat and humidistat to control temperature and humidity at the room level. Temperature and humidity are continuously monitored and alarms alert personnel to excursions from defined temperature or humidity ranges. Animal care technicians record high and low temperatures and humidity daily on a room log sheet using an electronic digital thermometer/humidistat.
Wild animals	No wild animals were used.
Reporting on sex	All studies used both genders to exclude gender-specific variation.
Field-collected samples	This study did not use field-collected samples.
Ethics oversight	All animal studies were performed in accordance with the recommendations in the Guide for the Care and Use of Laboratory Animals (the Guide) of the National Institutes of Health. Animal protocols were approved on an annual term by the Institutional Animal Care and Use Committee (IACUC) at St. Jude Children's Research Hospital.

Note that full information on the approval of the study protocol must also be provided in the manuscript.

Plants

Seed stocks	*Report on the source of all seed stocks or other plant material used. If applicable, state the seed stock centre and catalogue number. If plant specimens were collected from the field, describe the collection location, date and sampling procedures.*
Novel plant genotypes	*Describe the methods by which all novel plant genotypes were produced. This includes those generated by transgenic approaches, gene editing, chemical/radiation-based mutagenesis and hybridization. For transgenic lines, describe the transformation method, the number of independent lines analyzed and the generation upon which experiments were performed. For gene-edited lines, describe the editor used, the endogenous sequence targeted for editing, the targeting guide RNA sequence (if applicable) and how the editor was applied.*
Authentication	*Describe any authentication procedures for each seed stock used or novel genotype generated. Describe any experiments used to assess the effect of a mutation and, where applicable, how potential secondary effects (e.g. second site T-DNA insertions, mosiacism, off-target gene editing) were examined.*

Flow Cytometry

Plots

Confirm that:

- [x] The axis labels state the marker and fluorochrome used (e.g. CD4-FITC).
- [ ] The axis scales are clearly visible. Include numbers along axes only for bottom left plot of group (a 'group' is an analysis of identical markers).
- [x] All plots are contour plots with outliers or pseudocolor plots.
- [x] A numerical value for number of cells or percentage (with statistics) is provided.

Methodology

Sample preparation	For surface staining, samples were washed with and stained in PBS (Lonza) with 1% FBS (HyClone).
Instrument	BD FACS Canto.
Software	FlowJo (vIO) and BD FACSDiva.

Cell population abundance	Cells were analyzed for viability prior to flow cytometry with trypan blue exclusion with a 90% viable threshold for analysis. Cell permeating viability live/dead Aqua or DAPI live/dead was also used during flow cytometric analyses.
Gating strategy	1) Lymphocyte gate FSC vs SSC<br>2) Singlets: Doublets were excluded using FSC-H vs FSC-A as cells displaying double the signal of singlets.<br>3) Live/Dead Aqua or DAPI negative<br>4) Gating examples are provided as Supplementary Figure 19.

☒ Tick this box to confirm that a figure exemplifying the gating strategy is provided in the Supplementary Information.
